# Functional connectivity analysis in EEG source space: The choice of method

**DOI:** 10.1371/journal.pone.0181105

**Published:** 2017-07-20

**Authors:** Elham Barzegaran, Maria G. Knyazeva

**Affiliations:** Laboratoire de recherche en neuroimagerie (LREN), Department of Clinical Neurosciences, Centre Hospitalier Universitaire Vaudois and University of Lausanne, Lausanne, Switzerland; University of British Columbia, CANADA

## Abstract

Functional connectivity (FC) is among the most informative features derived from EEG. However, the most straightforward sensor-space analysis of FC is unreliable owing to volume conductance effects. An alternative—source-space analysis of FC—is optimal for high- and mid-density EEG (hdEEG, mdEEG); however, it is questionable for widely used low-density EEG (ldEEG) because of inadequate surface sampling. Here, using simulations, we investigate the performance of the two source FC methods, the inverse-based source FC (ISFC) and the cortical partial coherence (CPC). To examine the effects of localization errors of the inverse method on the FC estimation, we simulated an oscillatory source with varying locations and SNRs. To compare the FC estimations by the two methods, we simulated two synchronized sources with varying between-source distance and SNR. The simulations were implemented for hdEEG, mdEEG, and ldEEG. We showed that the performance of both methods deteriorates for deep sources owing to their inaccurate localization and smoothing. The accuracy of both methods improves with the increasing between-source distance. The best ISFC performance was achieved using hd/mdEEG, while the best CPC performance was observed with ldEEG. In conclusion, with hdEEG, ISFC outperforms CPC and therefore should be the preferred method. In the studies based on ldEEG, the CPC is a method of choice.

## Introduction

Cognitive functions are implemented via coordinated activity of the neural modules distributed in the brain [1, 2]. The coordination of modular activity is analyzed within the framework of a concept of the functional connectivity (FC) [3–5]. Among various methods for measuring the FC, electroencephalography- (EEG-) based techniques are unique in that they provide tools to evaluate the FC dynamics on a millisecond time scale inherent in cognitive processes. Some of these techniques estimate synchronization of distributed EEG signals recorded from the head surface [6, 7].

However, well-known limitations of this method include the lack of information about locations of the brain sources of EEG together with the signal mixing owing to the volume conductance and reference electrode, making interpretation of the sensor-space synchronization measures problematic [8–10]. Several strategies have been proposed to minimize the effect of volume conductance including estimation of FC from surface Laplacian [11], employment of FC measures robust to this effect (e.g., [12]), or estimation of FC limited to its changes in experimental contrasts [8]. However, such approaches can neither completely eliminate the effects of volume conductance, nor localize the EEG sources related the FC dynamics.

To overcome these limitations, source EEG has been used for the FC analysis [13, 14]. These methods can be divided into two main groups. One group, based on a biophysical generative model, which describes how the neural source dynamics and interactions can produce scalp EEG [15, 16], aims at direct estimation of the FC between sources from the sensor-space data and requires prior assumptions on the network structure.

The other group of methods, which exploit a two-step procedure, is model-free and does not need any assumptions on the network structure. In the first step, source signals are determined using an inverse solution. The latter can be based on distributed source models (Weighted Minimum Norm (WMN), Low Resolution Electromagnetic Tomography (LORETA), Local Auto Regressive Averaging (LAURA), etc.) or on dipolar source models. Different approaches can be applied prior to dipole fitting to separate sources of activity and to determine the number of dipoles [17–20]. In the second step, the FC is estimated between distributed or dipole sources ([21–23], for review see [24]).

These techniques provide an acceptable accuracy if applied to the high-density EEG (hdEEG) [17]. However, in clinical neuroscience, the mid-density (mdEEG) or low-density EEG (ldEEG) is frequently used, thus limiting the application of both groups of methods. A new technique of the cortical partial coherence (CPC) analysis, which reportedly can be used even with ldEEG, has been proposed by [25]. Its allegedly high potential for estimating the FC with ldEEG attracted our attention. The CPC method estimates the source FC through the partial coherence matrix of the sensor-space EEG. Unfortunately, the authors tested the performance of the method by a simple simulation of ldEEG, leaving out of consideration different signal-to-noise ratios (SNR) and source locations, which can strongly influence the accuracy of estimations.

Because of the strong interest in the FC concept in clinical neuroscience, where ldEEG is being routinely used, here we analyze the performance of the CPC method in comparison with the conventional two-step inverse-based source FC (ISFC) strategy using ldEEG (18 sensors), mdEEG (61 sensors), and hdEEG (110 sensors) in our simulations. To comprehensively consider the factors that can affect the accuracy of FC estimation, we implemented two simulations. In the first one, we addressed the accuracy of source localization, which can affect the FC estimation by the ISFC method and its interpretation. To this end, we modeled a single oscillatory time series as a source with varying location. In the second simulation, we modeled two synchronized time series, one of which had fixed and the other one, varying location. In both cases, we also varied SNR and localized EEG sources by means of different inverse solutions.

## Methods

### Source FC analysis

**Fig 1:**
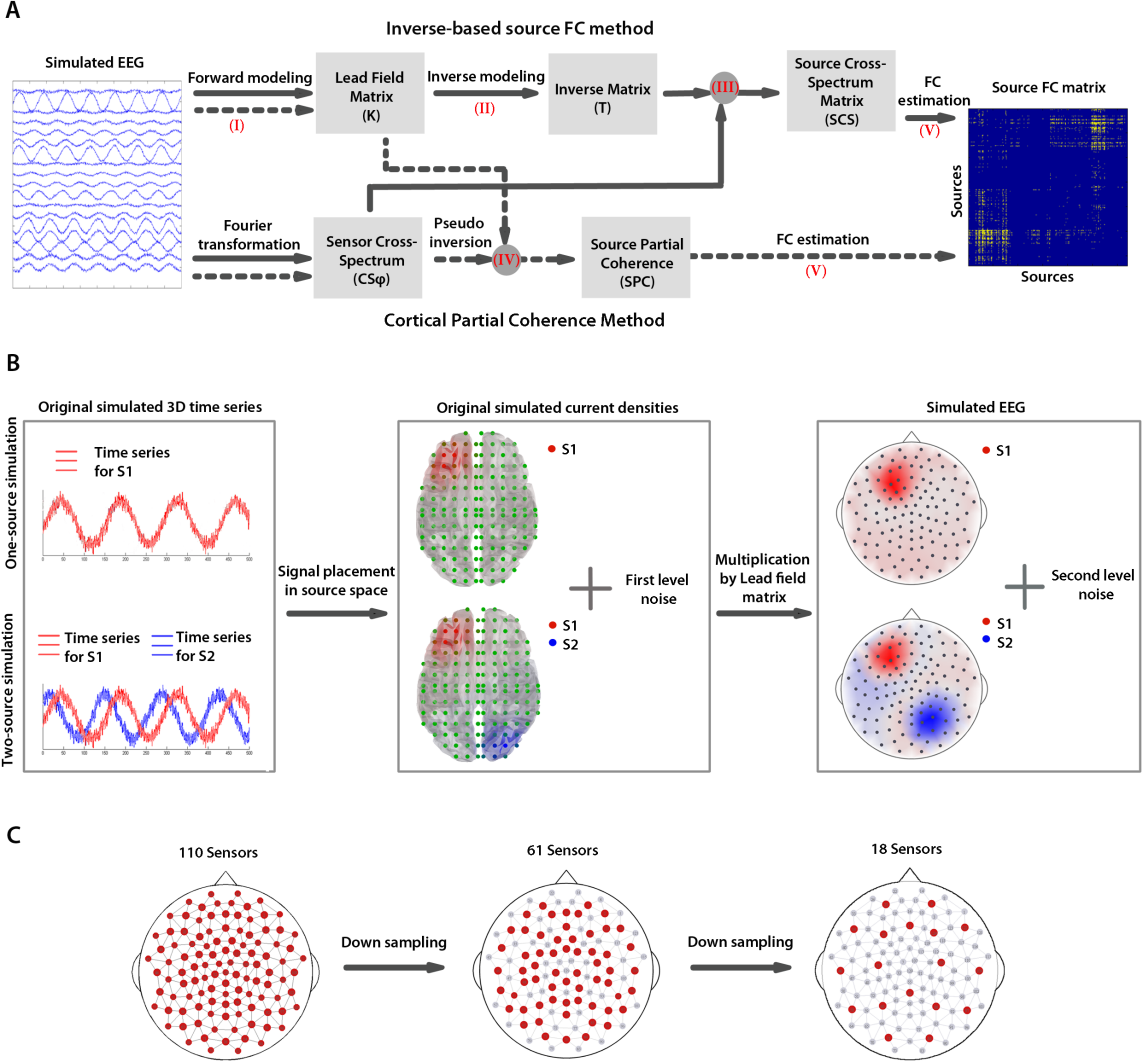
Methods for source FC estimation and EEG simulations. ***A*.** The block diagram represents principal steps for the source FC analysis by means of the ISFC (solid arrows) and CPC (dotted arrows) methods. Arrows indicate the steps of the analyses; the numbers in brackets refer to the paragraphs of the Method section describing these steps. The gray circles refer to the steps with more than one input. The gray rectangles represent the input/output of analysis steps. **B**. The procedures for one- and two-source EEG simulations are presented. First, one and two oscillatory time series were generated for one and two source simulations, respectively (left). Then, source current densities were generated by placing these signals in the source grid and adding the first level noise (middle). Finally, the source current densities were multiplied by the lead field matrix to generate the sensor-level signal, to which the second-level noise was added. **C.** Sensor layouts with 110, 61, and 18 sensors (in red) that were used in the simulations.

In order to compare the accuracy of the ISFC and CPC methods, we applied them to simulated EEG signals with known parameters (Fig 1). Specifically, for simulated EEGs we determined ***(I)*** forward solution for both methods; ***(II)*** inverse solution for ISFC; ***(III)*** source cross-spectrum (SCS) matrix for ISFC; ***(IV)*** source partial coherence (SPC) matrix for CPC; and ***(V)*** the source FC based on CSC and SPC matrices. The details of the steps ***(I)–(V)*** are explained in the following paragraphs.

***(I)*** *The* forward solution estimates how cortical currents generate scalp voltages. For the EEG data at a time point *t*, ($\varphi_{t}\epsilon R^{N_{C}\times 1}$), where R indicates real numbers, and *Nc* is the number of channels, the forward solution is defined as

$$\varphi_{t}=KJ_{t},$$ (1)

where $K\in R^{N_{C}\times {3.N}_{S}}$ is the lead field matrix, *N*_*S*_ is the number of sources. As the source current densities are estimated in three directions, one dimension of the lead field matrix is *3×N*_*S*_, and $J_{t}\in R^{{3.N}_{S}\times 1}$ is the source current density at a time point *t*.

The lead field matrix was estimated for 640 source points, uniformly distributed over a grid of cells ~12x12x12 mm^3^ in size, using Locally Spherical Model with Anatomical Constraints method [26]. The grid covered the gray matter of the Montreal Neurological Institute’s average brain [27].

***(II)*** The inverse solution estimates current source densities, which generate scalp potentials recorded by EEG. It is defined as

$$J_{t}=T\varphi_{t},$$ (2)

where $T\in R^{3.N_{S}\times N_{C}}$ is the inverse matrix. The estimation of this matrix is an ill-posed problem, which requires additional assumptions. Here, we used the WMN method [28, 29], which estimates sources by minimizing the solution power and using Tikhonov regularization [30]. This method applies weighting to the lead field matrix for balancing the estimations of superficial and deep sources. To test the consistency of our findings across different methods of source reconstruction, we added the LAURA [31] and LORETA [32] inverse solutions, the description of which is given in the S1 File. All the forward and inverse solutions have been implemented with Cartool toolbox [26].

***(III)*** To estimate the source cross-spectrum (SCS) matrix by the ISFC method, the sensor cross-spectrum matrix (${CS}_{\varphi }(f,e)\in c^{N_{C}\times N_{C}}$, where c represents complex numbers, was calculated as

$${CS}_{\varphi }(f,e)=\frac{2}{df}\varphi_{f,e}{\varphi_{f,e}}^{*},$$ (3)

where $\varphi_{f}\in c^{N_{C}\times 1}$ is the Fourier transformation of the EEG signal at an epoch *e* and frequency *f*, *df* is the frequency resolution, and superscript ‘*’ represents complex conjugate. Then, the source cross-spectrum matrix $SCS(f,e)\in c^{{3.N}_{S}\times 3.N_{S}}$ at frequency *f* and epoch *e* was calculated as

$$SCS\left(f,e\right)=T\;CS_{\varphi }\left(f,e\right)\;T^{'}.$$ (4)

***(IV)*** The source partial coherence $SPC(f,e)\in c^{{3.N}_{S}\times 3.N_{S}}$ matrix at frequency *f* and epoch *e* for CPC method was calculated as

$$SPC(f,e)=K'\;CS_{\varphi }{\left(f,e\right)}^{+}\;K,$$ (5)

where K is the lead field matrix calculated from (1), CS_φ_ is the sensor cross-spectrum defined in (3), and ‘+’ represents Moore-Penrose generalized inverse.

***(V)*** Various methods of FC estimation take into account different aspects of dependences between signals including linear, nonlinear, or information-based, as well as directed or undirected relationships and calculate the FC in frequency or time domains [7, 33–36]. We used an undirected linear frequency-domain measure of lagged dependence [37] that describes the lagged components of functional interactions (LFC). The LFC was estimated for each source pair based on SPC and SCS matrices. If we consider two sources *X* and *Y*, then the LFC at a frequency *f* and epoch *e* is calculated as

$${LFC}_{XY}=1-\frac{\left\{\left|\left(\begin{array}{cc} S_{YX} & S_{YX}\\ S_{XY} & S_{XX} \end{array}\right)\right|/\left|\left(\begin{array}{cc} S_{YY} & 0\\ 0 & S_{XX} \end{array}\right)\right|\right\}}{\left\{\left|Re(\begin{array}{cc} S_{YX} & S_{YX}\\ S_{XY} & S_{XX} \end{array})\right|/\left|Re(\begin{array}{cc} S_{YY} & 0\\ 0 & S_{XX} \end{array})\right|\right\}},$$ (6)

where |M| indicates the determinant of a complex-valued matrix M, Re(M) is the real value of M, S_XY_(f,e) represents the element in the row X and column Y of the SCS(f,e) or SPC(f,e) matrices (for ISFC and CPC methods, respectively). Then, the LFC values, which ranged between 0 (for two uncorrelated time series) and 1 (for two identical time series), were averaged over 10 epochs.

### EEG simulations

The first step in the source FC estimation with the ISFC method is to calculate the source cross-spectrum matrix. The accuracy of an inverse solution in localizing sources of EEG can affect this matrix and the following FC estimations. Therefore, initially, we evaluated the accuracy of source localization in the ISFC method using a single-source simulated EEG signal with a varying location in the source grid and with different SNRs (Fig 1B). Owing to the direct calculation of source partial coherence, in the CPC method, source current densities are not estimated and therefore the localization error of the method cannot be evaluated. To evaluate the accuracy of the FC estimation in the ISFC and CPC methods, we simulated EEG signals from two interacting sources located in different cortical regions and having different SNRs.

For the single oscillatory source, we generated an 8-Hz sinusoid time series with 10000 time samples at a sampling frequency of 1000 Hz. We placed this signal in the source grid using a spatial 3D Gaussian function with diagonal covariance matrix (SD_O_ = 10 mm) and centered it on each of 640 grid cells. In the two-source simulation, for the first source (S1), we used the same signal as described above, whereas, for the second source (S2), we generated a sinusoid signal with the same frequency and relative phase of 90° to the signal S1. The S1 location was fixed in the center of the middle frontal gyrus (MFG), whereas S2 had varying location in the source grid with a minimum distance of 20 mm from S1. To place these signals in the source space, we used a Gaussian function with the diagonal covariance matrix as in the single source simulation. In both simulations, we added zero-mean Gaussian noise that represented biological noise to each source potential.

To generate EEG time series, we multiplied the source current densities by the lead-field matrix at each time point according to (1). Finally, to approximate the measurement noise, we added zero-mean Gaussian noise to the simulated sensor potentials. The SNR for the biological and measurement noise levels was defined as

$$SNR=10\;\log\left(\frac{Power_{simulated\;signal}}{Power_{noise}}\right)\;dB.$$ (7)

The SNR varied between 0 and 20 dB.

We simulated the one-source and two-source EEGs for the sensor arrays representing hdEEG (110 sensors), mdEEG (61 sensors) and ldEEG (18 sensors). To obtain more sparse arrays, the 110-channel set of sensors was down-sampled to 61 and further to 18 sensors, uniformly distributed over scalp (Fig 1C).

### Evaluation of ISFC and CPC methods

To evaluate the localization error of WMN inverse solution, used in the ISFC method in one-source simulation, we used two error measures. The first one—the error distance (ED)—calculates a Euclidean distance between the original source location and the maximum of the reconstructed power current density. This error has been extensively used to evaluate the performance of several methods in localizing EEG sources [17, 38, 39]. To obtain the second one—error standard deviation (ESD)—the estimated standard deviation of a Gaussian function centered on the maximum of reconstructed power current density (SD_R_) was divided by the same measure of original power current density (SD_O_), i.e., ESD = SD_R_/SD_O_. The ED and ESD were calculated for each SNR and sensor set.

In order to compare the performance of the ISFC and CPC methods in the two-source simulation, the FC error (FCE) for the S1-S2 pair was defined as

$$FCE=\frac{\sum_{n=1}^{p}\left|LFC_{s1}\left(n\right).d_{s2}\left(n\right)\right|}{\sum_{n=1}^{p}\left|d_{s2}\left(n\right)\right|},$$ (8)

where *d*_*s*2_(*n*) is the distance between S2 and source *n*, and *LFC*_*S1*_*(n)* is the estimated LFC between S1 and source *n*. For calculation of the FCE, the *LFC*_*s1*_ values were normalized over all *p* sources of the source grid, so that the maximum was set to 1 and minimum, to 0. The FCE calculates the average FC error for source points, weighted by their distances from the S2. It has a minimum value of about 0 and maximum value of 1.

## Results

We obtained similar results for the WMN, LORETA, and LAURA methods in one- and two-source simulations. Here we present the WMN results, whereas the comparative analysis of the effects of these methods on the FC estimation can be found in the S1 File.

**Fig 2:**
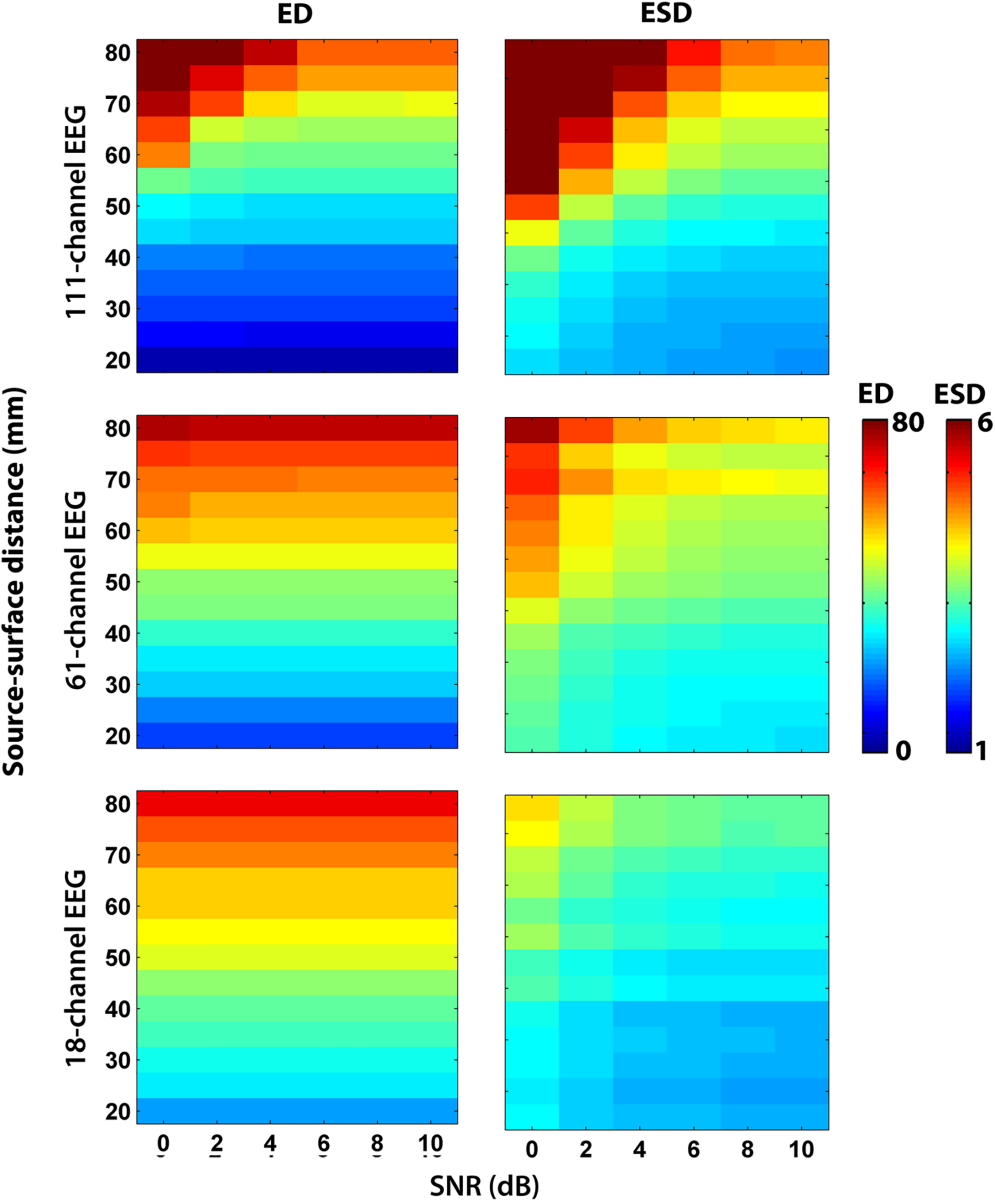
Localization errors of WMN method. The error distance (ED) and error standard deviation (ESD) for each sensor array is presented with a heat map as a function of SNR and source distances. The color bars on the right show the ED and ESD scales.

For the analysis of one-source EEG simulations, we considered superficial sources located at 20–40 mm from the head surface, medium ones, at 40–60 mm, and deep sources at a distance of 60–80 mm. The deep sources reconstructed with the WMN had higher ED and ESD than superficial sources with all SNRs (Fig 2). The ED for superficial sources were on average, around 11 mm, which is equal to the displacement by 0-to-1 unit of the 12x12x12 mm^3^ source grid for hdEEG. For EEG arrays with 61 and 18 sensors, this error was about 20–30 mm, i.e., equal to the displacement by 2–3 grid units. For the sources at medium distances, the ED for hdEEG was about 30 mm (2–3 grid units displacement) and 40–50 mm for mdEEG and ldEEG (the displacement by > 3 grid units). The deep sources could not be localized, since the ED > 50 mm for all sensor arrays (the displacement by ≥ 5 grid units).

**Fig 3:**
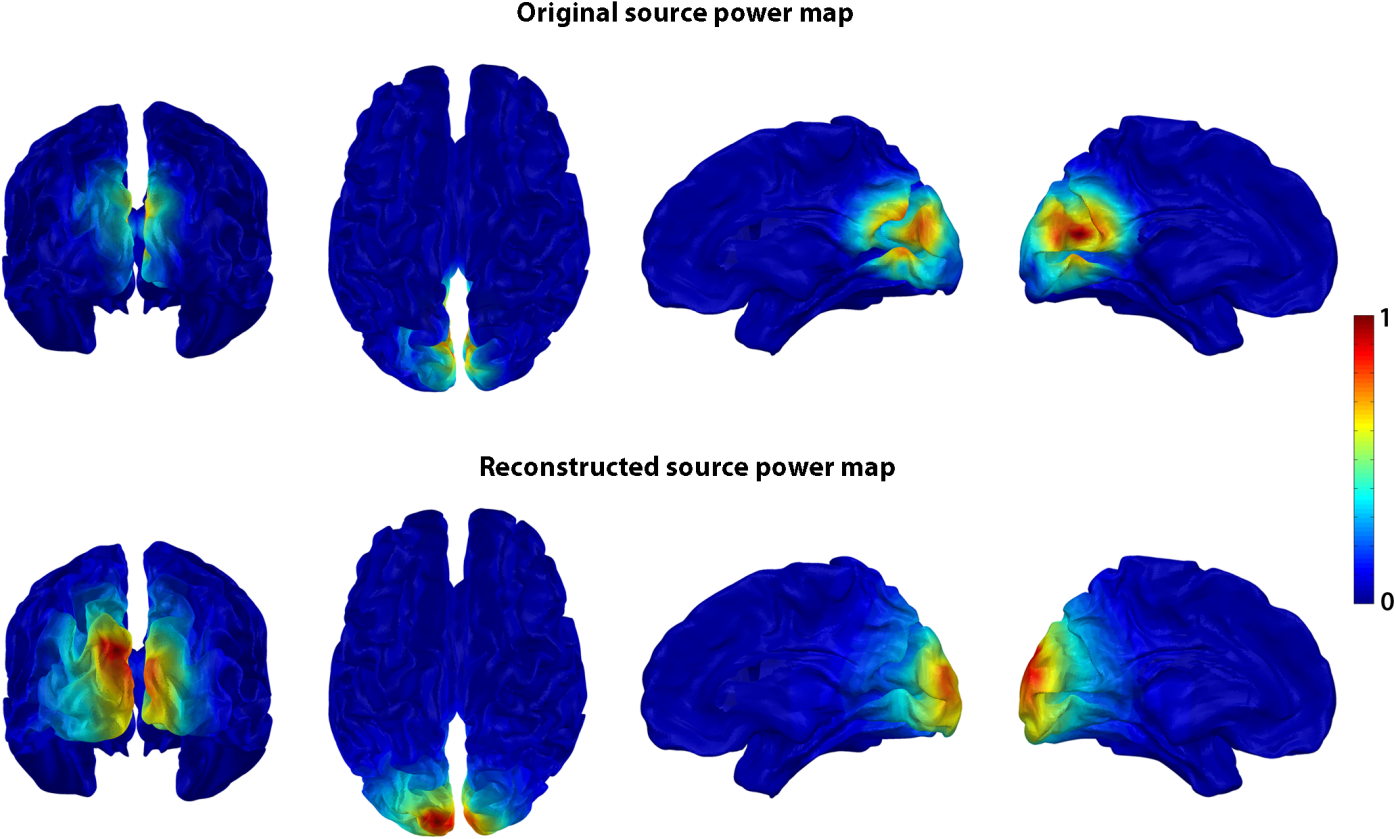
Source localization error for deep sources using WMN inverse solution. The original source power map before forward modeling (top row), and the reconstructed source power map after inverse modeling (bottom row) are presented for one-source simulation with 111-sensor EEG array and SNR = 4 dB. The source power maps are rendered on the MNI average brain and presented in four views (the posterior and top views of the whole brain and the mid-sagittal views of the right and left hemispheres). In this simulation, S1 was originally centered in the cuneus, at a distance of 45 mm from the scalp surface. The reconstructed current sources were localized more superficially in the superior occipital gyrus at a distance of 25 mm between the source with maximum power and the scalp surface. The color bar on the right shows the normalized power scale.

The ESD values showed a 2-3-time increase of smoothness for superficial sources with all sensor densities, whereas for all other sources they showed more than a 4-time increase with hdEEG and mdEEG and a 2-3-time increase with ldEEG. An example of inaccurate source reconstruction for the source located at a distance of 50 mm from head surface is presented in Fig 3. The increase of smoothness can be clearly seen in this case (ED = 24 mm or 2 grid units and ESD = 3.6).

**Fig 4:**
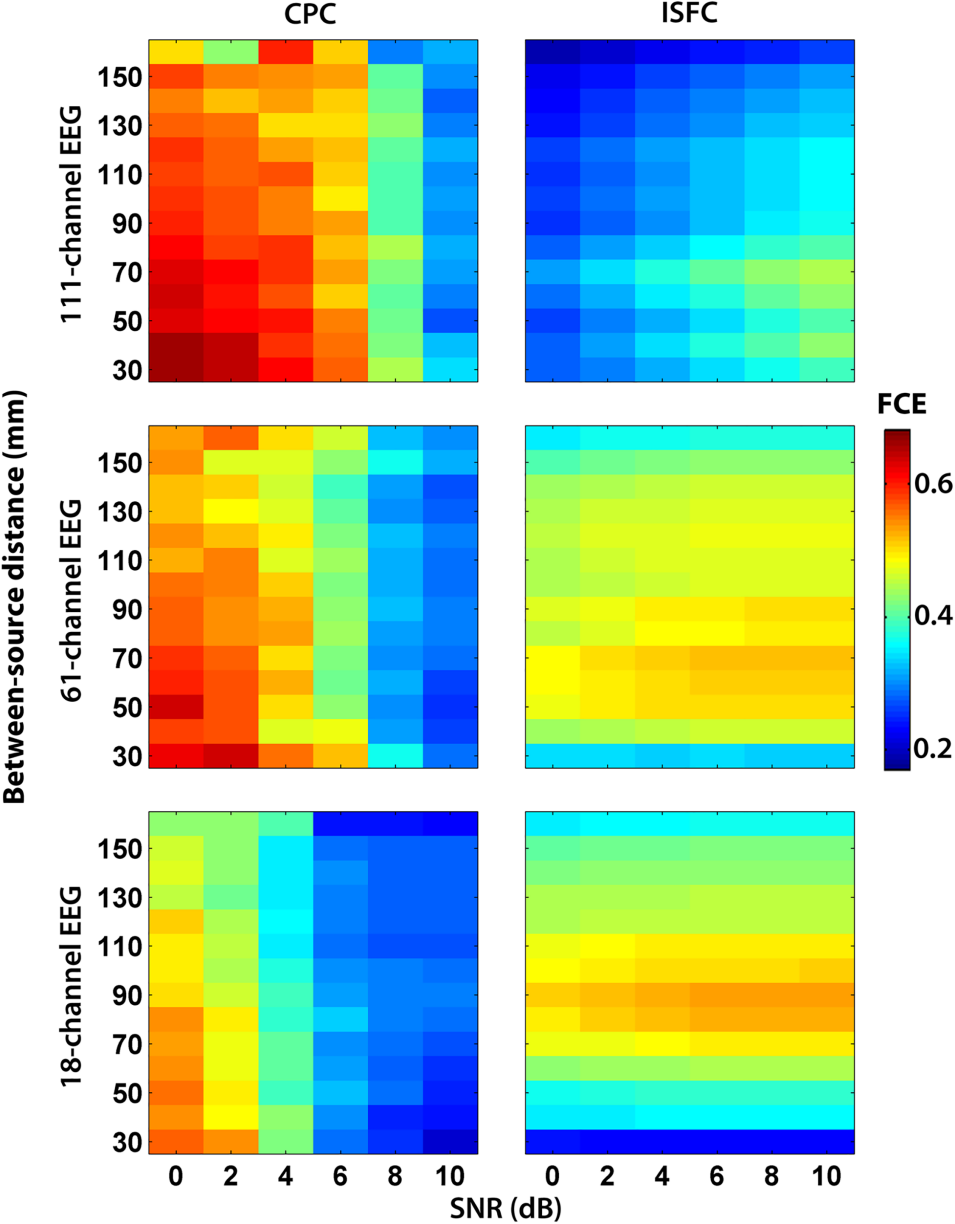
Functional connectivity error (FCE) of CPC and ISFC methods. The FCE is presented with a heat map as a function of SNR and between-source distances for each sensor array. The color bar on the right shows the FCE scale.

The two-source simulations indicated that the FC accuracy depended on the SNR and the sensor density (Fig 4). For the ISFC method, the FCE decreased with the increasing number of sensors. In addition, the FCE increased with increasing SNR. Therefore, this method performed best with relatively weak sources reconstructed from hdEEG.

The CPC method was highly affected by the SNR (Fig 4). At low SNRs (< 6 dB), it was less accurate than the ISFC method. However, for EEG simulated with high SNR (> 6 dB), the CPC performance was superior to that of the ISFC for mdEEG and ldEEG. For all tested SNRs, the CPC method performed best with with ldEEG.

**Fig 5:**
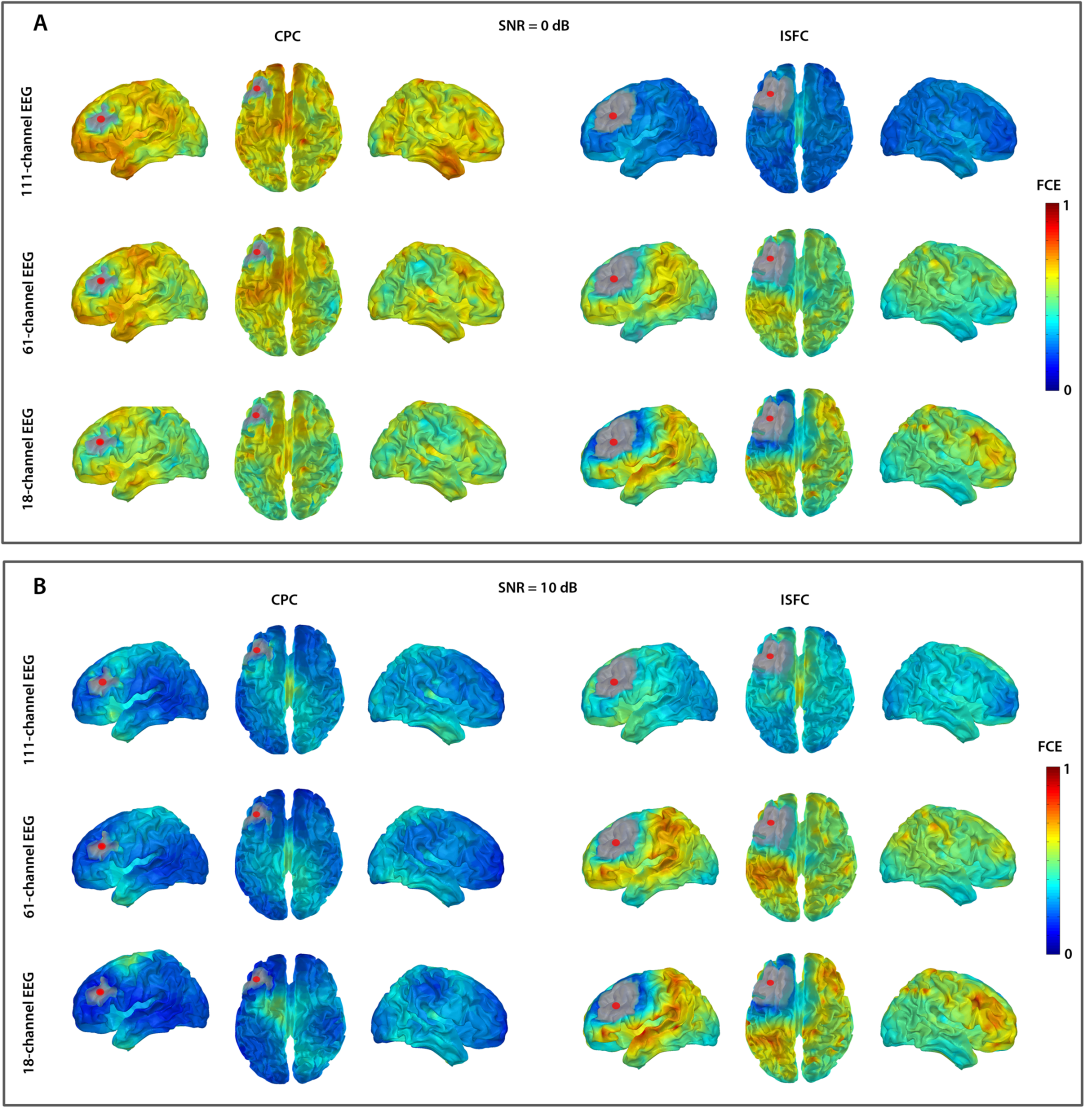
Topography of functional connectivity error (FCE) for CPC and ISFC methods. The FCE maps for the three sensor arrays at SNR = 0 dB (A) and SNR = 10 dB (B) are presented. The FCE for the S2 locations that cover the entire source grid (except the vicinity of S1 shown in gray) is rendered on the average MNI brain and presented in three views (the left hemisphere, the top view of the whole brain, and the right hemisphere). The color bars indicate the scaling of FCE.

The performance of both methods also depended on the between-source distance, the mean value of which was 90 mm. If the two sources were located at a close distance from each other (< 90 mm), the FCE was higher than if there was a long distance between them (> 90 mm) (Fig 3). We observed this effect with all the SNRs and sensor arrays. For example, when the fixed source (S1) was placed in the middle frontal gyrus (MFG), we obtained the smallest FCE—if the source S2 was located in the posterior cortices, i.e., at the longest distance from MFG (Fig 5).

**Fig 6:**
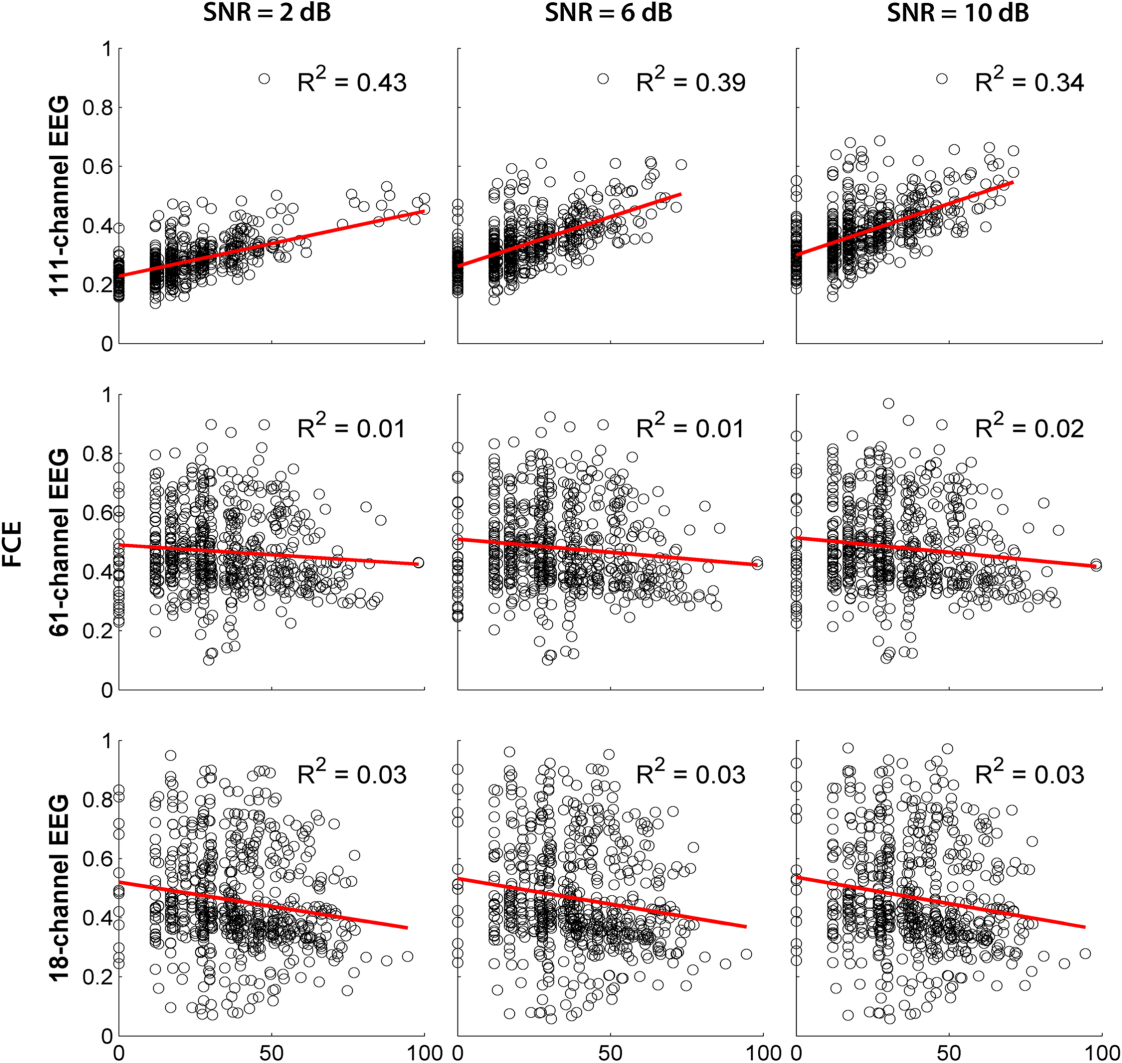
Functional connectivity error (FCE) of ISFC method as a function of source mislocalization. The scatterplots of FCE as a function of ED are shown for the three sensor arrays at three levels of SNR. Each data point (a circle) on the scatterplots represents the FCE and ED of a source. The regression of the FCE as a dependent variable on the ED as an explanatory variable is shown with red lines. For all R-squared (R^2^) values, P < 0.01.

Finally, to check whether the FCE (for the FC assessed with the ISFC method) depends on the error of source localization (ED) in the WMN method, we applied a linear regression analysis (Fig 6). As can be seen from the values of R-squared presented in this figure, in hdEEG, around 40% of the FCE variance can be explained by source mislocalization, while, in md/ldEEG, this value is less than 5% against much higher FCE values. This was true for all the tested SNR levels (0–10 dB with a step of 2 dB).

## Discussion

The studied factors that affect the accuracy of source FC estimation by means of the CPC and ISFC methods can be summarized as follows. The performance of the methods depends on the number of EEG sensors, on the source depth and between-source distances, and on the SNR level. For both methods, the increase of the source depth deteriorates the accuracy of FC estimations owing to the decreased accuracy of source localization and size. The FC can be more precisely estimated between distant sources than between close ones, independent of the method used. For the ISFC method, specifically, the FC accuracy increases with increasing sensor density, but not with SNR. In contrast, the performance of the CPC method improves with increasing SNR, but mildly declines with increasing the number of sensors.

### Effects of source localization

The FC analysis in the brain source space should answer at least two questions: how the FC is changing in the condition or the group in question, and what structures are responsible for these changes. Our findings demonstrate that the mislocalization of EEG sources is likely to affect both estimation and interpretation of the FC.

The one-source simulation showed that the localization error (ED) of WMN and other inverse solutions conventionally used in the ISFC method depends on the source depth, the SNR, and sensor density, thus confirming previous results [38, 40, 41]. Deep sources cannot be localized accurately with any EEG density. For instance, in spite of weighting used in the WMN, this method has the tendency to map the signal of deep sources to the superficial ones (Fig 3). The same tendency is also characteristic for other methods (Fig A in S1 File). The failure of these inverse solutions to provide accurate information about source depth results in underestimating the FC between deeper sources and other regions, and in overestimating the FC between spurious superficial sources propagated from strong deep sources to the scalp surface. The regression analysis confirmed strong relationship between the ED and FC estimation errors (FCE), especially, for hdEEG, where the FC measures are more reliable, pointing to the important role of source localization for the FC estimation.

A possible solution for minimizing the effect of inaccurate source localization is choosing an appropriate cortical parcellation, i.e., fine subdivision of superficial regions and relatively coarse subdivision of deeper ones [42]. Furthermore, applying the new methods that include additional priors from structural and functional MRI would improve source localization of EEG/MEG for the cortical and subcortical regions [43, 44].

The increased spatial extent of reconstructed sources, especially of deep ones shown here in the one-source simulation can be also attributed to the WMN limitations in localizing deep sources. Because of the effect of volume conductance, the signal of a deep source propagates to many sensors and therefore is being reconstructed as enlarged and more superficial than the original one (Fig 3). The increased smoothness of the reconstructed sources highlights the importance of selecting an appropriate FC measure. Specifically, instantaneous indexes of FC would overestimate the interactions between close sources, providing undesirable consequences for further analysis. For instance, in the network-based analysis, the local connectivity measured with clustering coefficient may be overestimated [23, 34]. Measures immune to the instantaneous interactions of signals e.g., lagged indexes of FC [12, 37], as well as new methods of EEG source estimation, which reduce the ESD error [45], can be recommended.

### Effects of sensor density

The studies based on the simulated and recorded EEG showed that the between-sensor distance of 2–3 cm, corresponding to approximately 128 channels (hdEEG), is required for the accurate spatial sampling [46, 47]. However, [17] showed that various inverse solutions provide adequate source localization accuracy starting with mdEEG (≥ 60 channels). In [41], Sohrabpour with colleagues were able to refine that both localization and effective connectivity errors decrease with increasing the number of channels, although this effect is small if the latter is more than 64.

In agreement with these findings, we have shown the improved performance of the ISFC method with increased sensor density providing the same SNR [39, 48, 49]. In the hdEEG simulations, the ISFC method outperformed the CPC technique at different SNR levels. In contrast, the performance of the CPC method was superior to the ISFC one with ldEEG, once the SNR was high; however, it deteriorated with increasing sensor density. A similar effect, i.e., the disadvantage of the increasing the number of sensors for the methods that rely on an accurate estimation of the lead field matrix was discussed in [34]. This can be one aspect of the explanation for worsening the performance of the CPC with hdEEG.

### Effects of between-source distance

In the two-source simulation, the accuracy of FC estimation improved by increasing the between-source distance in both CPC and ISFC methods under all tested SNRs and sensor densities. This effect can be explained by the above discussed localization and spatial smoothing errors owing to volume conductance and by imprecise source un-mixing by means of the inverse solution. The longer the distance between sources, the less the superposition of the signals in the sensor space and more successful the inverse solution in un-mixing source signals. [8, 23] used simulations to show that although source estimation can reduce the artificial synchronization detected in sensor space, residual spurious FC can be present in the reconstructed source space. The effect of the between-source distance on the FC can be minimized by selecting a robust FC estimator, appropriate cortical parcellation, and inverse solution.

### Effects of SNR

We used SNR for defining two levels of noise, at the sensor and source level. The noise at the sensor level can originate, for instance, from muscle activity or eye movements. Contamination of EEG signal with such noise can result in the underestimation of FC [50]. The noise at the source level can also influence the estimation of FC. In particular, high-amplitude uncorrelated noise dominating the signal from synchronized sources would result in the underestimation of FC as discussed in [8]. Recently similar effects of noise on source localization and effective connectivity have been shown [41, 51].

In our analysis, the higher SNR results in a more accurate FC estimation with the CPC method and a slightly less accurate FC estimation with the ISFC method, all other factors being equal. The high sensitivity of the CPC method to the SNR level is apparently owed to the lack of regularization in the process of source FC estimation, as the source partial coherence is calculated directly from sensor coherence. Regularization, being an important part of inverse solution algorithms, serves to model the data in the presence of noise [52]. Several methods may be applied for reducing the noise of different origin in EEG signal, including blind source separation techniques based on PCA or ICA [53]. Nonetheless, if the noise level is significant, which might be the case for real EEG, the FC estimations by the CPC method can be inaccurate.

At high SNRs, the performance of the CPC method surpasses the ISFC one, all other factors being equal. This is likely attributable to partializing the coherence values that reduces the effects of volume conductance [25]. In contrast, the ISFC method cannot un-mix sources completely, if the amplitudes of synchronized sources are high compared to uncorrelated sources, as their signals are propagated in all directions because of volume conductance.

## Conclusion

The methods for source FC studies should be carefully chosen with regard to the most important factors that affect the FC measurements and are comprehensively analyzed and discussed here. In general, the ISFC method compared to the CPC one is a more accurate technique that is relatively immune to noise, given the high number of sensors used. Yet, for conventional ldEEG, the CPC method is an optimal choice, provided appropriate precautions are taken to ensure high SNR. In addition, independent of the method, the FC findings should not be over-interpreted considering the limitations inherent for deep and /or close sources.

## Supporting information

S1 File **(Fig A.) Error distance (ED) of ISFC method with WMN, LORETA, and LAURA inverse solutions**. The error distance (ED) for each sensor array and inverse solution is presented with a heat map as a function of SNR and source distance. The color bar on the right shows the ED scale in mm.
**(Fig B.) Functional connectivity error (FCE) of ISFC method with WMN, LORETA, and LAURA inverse solutions**. The FCE is presented with a heat map as a function of SNR and between-source distances (vertical axis) for each sensor array. The color bar on the right shows the FCE scale.
(DOCX)
