# Supplementary material for: Functional connectivity analysis in EEG source space: The choice of method
Source: PLoS One. 2017 Jul 20;12(7):e0181105. doi: 10.1371/journal.pone.0181105 (PMC5519059; doi:10.1371/journal.pone.0181105)
Supplement: S1 File — (Fig A.) Error distance (ED) of ISFC method with WMN, LORETA, and LAURA inverse solutions. The error distance (ED) for each sensor array and inverse solution is presented with a heat map as a function of SNR and source distance. The color bar on the right shows the ED scale in mm. (Fig B.) Functional connectivity error (FCE) of ISFC method with WMN, LORETA, and LAURA inverse solutions. The FCE is presented with a heat map as a function of SNR and between-source distances (vertical axis) for each sensor array. The color bar on the right shows the FCE scale. (DOCX) [file pone.0181105.s001.docx]

***S1 Supporting Information***

**Effect of inverse solution on ISFC method**

To consolidate the effects of various inverse solutions on the FC estimation, we implemented the ISFC method using the LOw Resolution Electrical Tomography (LORETA) [[1](#_ENREF_1)] and the Local Auto-Regressive Average (LAURA) [[2](#_ENREF_2)] solutions in addition to the Weighted Minimum Norm (WMN) [[3](#_ENREF_3)] solution described in the paper. These three methods have different assumptions for solving the ill-posed inverse problem. The WMN estimates distributed sources using Tikhonov regularization [[4](#_ENREF_4)] and minimizing the power of distributed sources, while balancing the estimation of superficial and deep sources with weightings. The LORETA normalizes the lead-field matrix to estimate deep sources and utilizes Laplacian operator to assess smoothed distributed sources. The LAURA applies constraints based on the biophysical laws of the electric field spread among neighboring sources. The forward solution for these inverse models was calculated for 640 distributed sources (see Methods for details) and the three inverse matrices were computed using Cartool toolbox [[5](#_ENREF_5)]. One- and two-source simulations were implemented for each method, and error distance (ED) and functional connectivity error (FCE) were calculated for different SNRs and sensor densities (See the Methods for details).

***One-source simulation.*** Under all SNRs and sensor densities, ED values were similar across the LORETA and LAURA, but slightly higher in the WMN, especially for deep sources and md/hdEEG (Fig A). The ED for superficial sources was 9-12 mm for all tested inverse solutions in hdEEG, which is equal to the displacement by 0-to-1 unit of the 12x12x12 mm^3^ source grid. For ld/mdEEG arrays, this error was about 20-30 mm, i.e., equal to the displacement by 2-3 grid units for all the methods.

For the sources at medium distances from the head surface, the ED was about 20-30 mm (2-3 grid units displacement) for hdEEG and 30-40 mm for md/ldEEG (the displacement by 3-4 grid units) with the LORETA and LAURA, while with WMN, it was ~30 mm for hdEEG and 40-50 mm (> 3 grid units displacement) for md/ldEEG. Neither of these methods could localize deep sources in md/ldEEG (ED > 50 mm, > 3 grid units). In hdEEG, the LORETA and LAURA had ED around 30-40 mm, if the SNR was high (> 4 dB), and > 50 mm (> 3 grid units), if the SNR was low, while the WMN had ED > 50 mm at all SNRs.


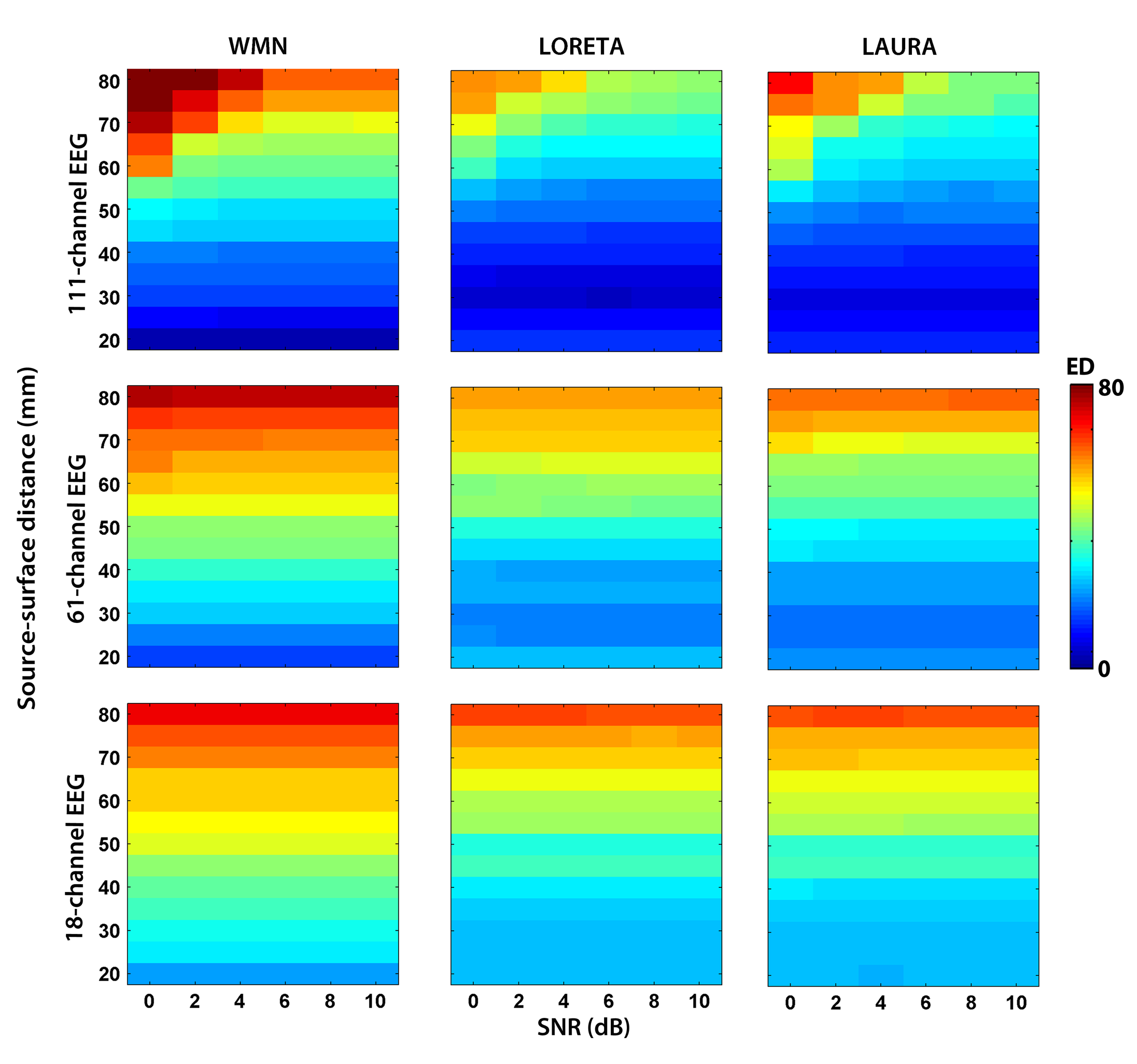


**Fig A. Error distance (ED) of ISFC method with WMN, LORETA, and LAURA inverse solutions**. The error distance (ED) for each sensor array and inverse solution is presented with a heat map as a function of SNR and source distance. The color bar on the right shows the ED scale in mm.

***Two-source simulation***. The three inverse solutions showed similar performance in the estimation of FC for all tested sensor arrays and SNRs. As Fig B. shows, the FCE decreases with increasing number of electrodes and between-source distance and increases with increasing SNR.

Therefore, all the tested methods of source modeling perform best for distant sources reconstructed from hdEEG. On the other hand, the source localization and FC estimation with the ISFC method based on mdEEG and, particularly, on ldEEG is less accurate irrespective of the inverse solution used (see the Results for other details).


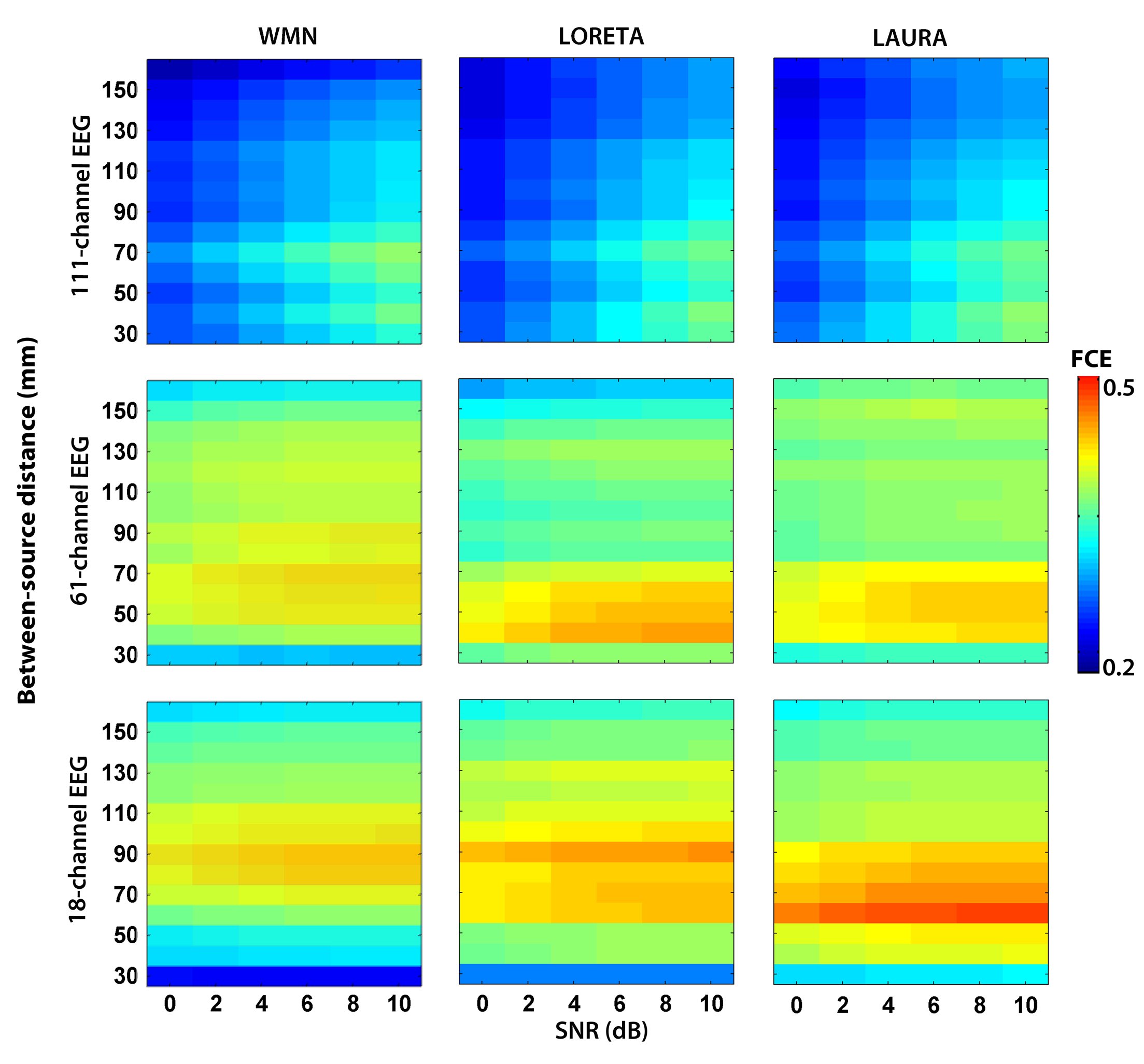


**Fig B. Functional connectivity error (FCE) of ISFC method with WMN, LORETA, and LAURA inverse solutions**. The FCE is presented with a heat map as a function of SNR and between-source distances (vertical axis) for each sensor array. The color bar on the right shows the FCE scale.

**References**

**1. Pascual-Marqui RD, Michel CM, Lehmann D. Low resolution electromagnetic tomography: a new method for localizing electrical activity in the brain. International Journal of psychophysiology. 1994;18(1):49-65.**

**2. de Peralta Menendez RG, Andino SG, Lantz G, Michel CM, Landis T. Noninvasive localization of electromagnetic epileptic activity. I. Method descriptions and simulations. Brain topography. 2001;14(2):131-7.**

**3. Fuchs M, Wagner M, Köhler T, Wischmann H-A. Linear and nonlinear current density reconstructions. Journal of Clinical Neurophysiology. 1999;16(3):267-95.**

**4. Tikhonov AN, Arsenin VY. Solutions of ill-posed problems. 1977.**

**5. Brunet D, Murray MM, Michel CM. Spatiotemporal analysis of multichannel EEG: CARTOOL. Computational intelligence and neuroscience. 2011;2011:2.**
